# Not Just Myocarditis: Mixed Connective Tissue Disease (MCTD) and Overlap Myositis With Anti-Ku Positivity in a Young Male With Shortness of Breath

**DOI:** 10.7759/cureus.72310

**Published:** 2024-10-24

**Authors:** Kawthar Alsulami, Julie D'Aoust

**Affiliations:** 1 Rheumatology, King Abdulaziz University Faculty of Medicine, Jeddah, SAU; 2 Rheumatology, University of Ottawa, Ottawa, CAN

**Keywords:** anti-ku antibodies, inflammatory myositis, overlap myositis, peri-myocarditis, scleromyositis

## Abstract

Mixed connective tissue disease (MCTD) is an autoimmune disorder characterized by high levels of anti-U1 ribonucleoprotein (RNP) antibodies and overlapping clinical features of autoimmune diseases, such as systemic lupus erythematosus (SLE), systemic sclerosis (SSc), and polymyositis (PM). Anti-Ku antibodies have been associated with overlap syndromes, which can present with symptoms such as Raynaud’s phenomenon, arthritis, and myositis.

A 19-year-old male athlete presented with myositis, notable for cardiac involvement. Diagnostic testing revealed elevated anti-RNP and anti-Ku antibodies, and a muscle biopsy indicated scleromyositis/overlap myositis. The patient was treated with high-dose corticosteroids, intravenous immunoglobulin (IVIG), rituximab, and mycophenolate mofetil, which led to significant improvement in muscle strength and cardiac function.

This case highlights the diagnostic complexity of MCTD when associated with positive anti-Ku antibodies, overlap syndromes, and cardiac involvement. Successful management emphasizes the importance of a tailored, multi-modal therapeutic approach.

## Introduction

Mixed connective tissue disease (MCTD) is a complex systemic rheumatic condition characterized by the presence of high levels of anti-U1 ribonucleoprotein (RNP) antibodies. MCTD shares clinical characteristics that are typical of several autoimmune illnesses, including systemic lupus erythematosus (SLE), rheumatoid arthritis (RA), systemic sclerosis (SSc), and polymyositis (PM) [1]. While myocarditis is not commonly observed as an initial manifestation, myositis is often a prominent characteristic of MCTD [2]. However, the process of diagnosing it can be challenging, particularly in cases where there are complicating factors, such as the use of myopathic medications.

Myositis-associated anti-Ku antibodies specifically target a heterodimer complex involved in DNA repair. Various autoimmune disorders, specifically SLE, SSc, and MCTD, are associated with these autoantibodies. Although anti-Ku antibodies have not been associated with a fixed clinical phenotype, patients frequently display symptoms such as Raynaud’s phenomenon, arthralgias, and muscle weakness. Anti-Ku antibodies have been identified in patients with overlap syndromes of inflammatory myopathy and a variety of autoimmune connective tissue diseases [3].

This case report describes a patient who presented with myocarditis and myositis. Ultimately, the patient was diagnosed with MCTD and overlap myositis with positive anti-Ku antibodies. Muscle pathology exhibited characteristics that were suggestive of an overlap myositis.

## Case presentation

**Figure 1:**
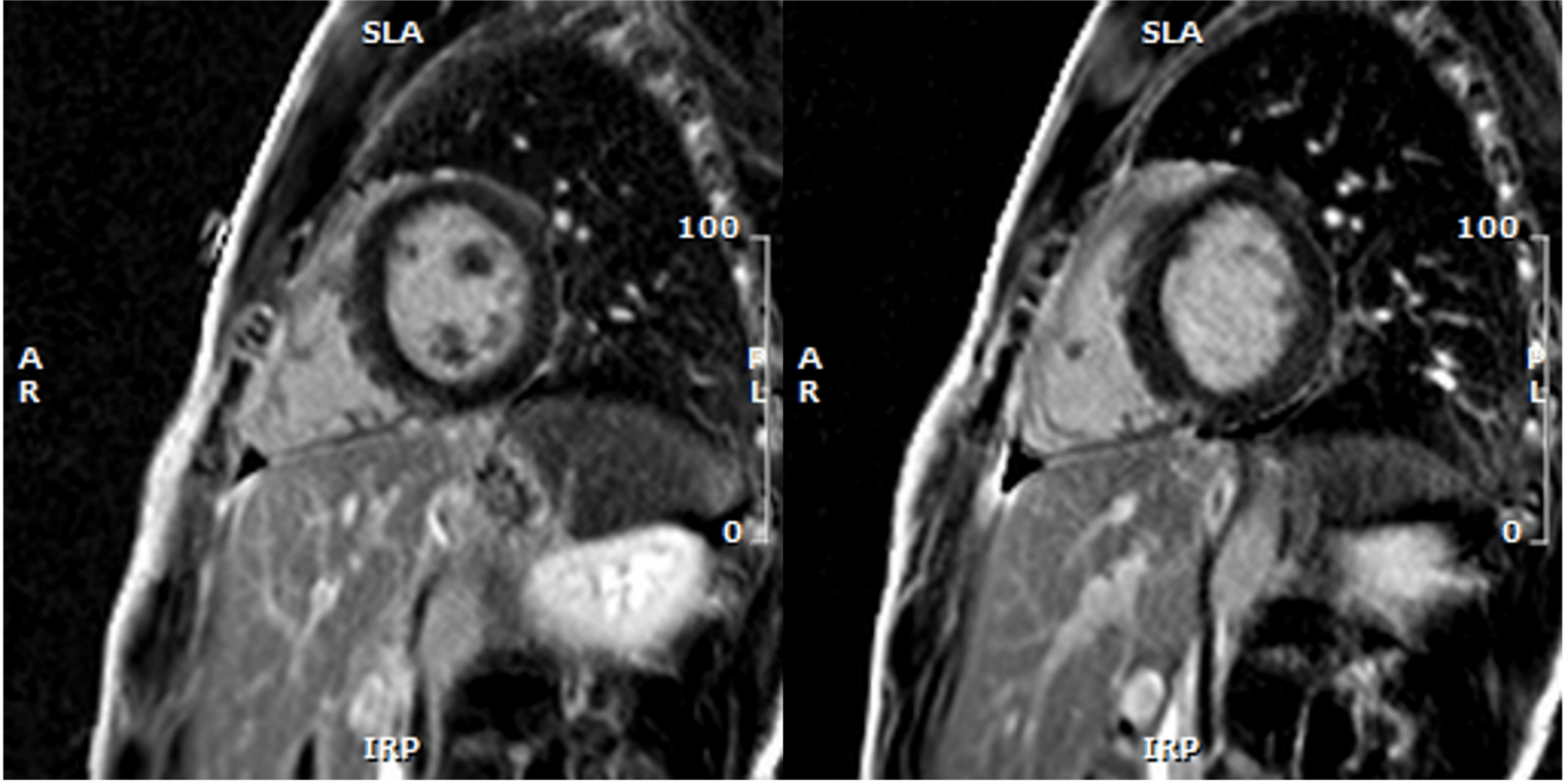
Cardiac MRI with gadolinium enhancement, demonstrating mild late enhancement at the inferior insertion point of the right ventricle into the septum. The elevated native T1 times and extracellular volume (ECV) values may indicate hyperemia and necrosis.

This case is of a 19-year-old, previously healthy, student-athlete, originally from South Africa. He was in his usual state of health until two months prior to presentation, when he began to experience progressive shortness of breath and chest discomfort at presentation. He had grade 3 dyspnea according to the Medical Research Council (MRC) scale, and his ability to participate in sports was significantly limited. The patient presented to the ER and was diagnosed with perimyocarditis on the basis of his symptoms and elevated troponin T values (223 ng/L, upper limit of normal 15 ng/L). Naproxen and colchicine were initiated by the emergency physician. After a period of two weeks, he returned with worsening chest heaviness, shortness of breath, overall fatigue, myalgia, as well as weight loss. The patient's troponin levels had increased twofold from the initial test to 673 ng/L. He was admitted to the cardiology department with suspected myocarditis based on his clinical presentation, elevated cardiac enzymes, and mild myocardial enhancement observed on MRI, as shown in Figure 1. A transthoracic echocardiogram demonstrated normal cardiac function, and no abnormalities in conduction were detected on the electrocardiogram.

Rheumatology was consulted to assess for an underlying autoimmune connective tissue disorder that might explain his presentation. During the assessment, the patient also reported generalized fatigue, which he initially attributed to the shortness of breath. He did not notice any difficulty getting out of bed and was able to ambulate normally. There were no reports of mucocutaneous symptoms, Raynaud's phenomenon, dysphagia, or gastroesophageal reflux disease (GERD). The clinical examination revealed mild muscle weakness in the symmetrical proximal upper and lower extremities, with a strength level of 4 in the deltoid and biceps muscles, and a plus-4 level in the gluteus maximus, medius, and quadriceps, based on the MRC Muscle Scale. The axial and distal muscles showed normal power. He was found to have less muscle bulk than expected and experienced tenderness to touch, primarily in the proximal, lower extremities. The cranial nerve examination was normal. We observed generalized hyporeflexia, where only reinforcements rated 1+ could achieve reflexes. No abnormalities on the sensory exam were detected. He exhibited bilateral elbow swelling with limited full extension but did not show synovitis in any other joints. The results of the nail capillaroscopy were unremarkable. There were no signs of hair loss, skin rashes, or skin tightness.

As shown in Table 1, the first blood test showed high levels of liver enzymes and creatine kinase (CK) at 24,349 U/L (the normal range is 54-320 U/L). Electromyography (EMG) revealed consistent and noticeable abnormalities, including fibrillations, positive sharp waves, and abnormal spontaneous activity. These abnormalities were observed in the left deltoid, left triceps, left vastus lateralis, and left tibialis anterior muscles. Additionally, myopathic changes were noted, such as patchy early increases in recruitment and occasional small amplitude/short duration motor unit potentials. Based on these findings, inflammatory myositis was considered the most likely cause. However, a consulting neurologist also identified colchicine neuromyopathy as a possible factor, prompting the discontinuation of colchicine. A muscle biopsy was subsequently requested. A CT chest was unremarkable. The serological profile revealed a significantly raised anti-nuclear antibody titer (ANA) of 1:2560, displaying a speckled pattern. Positive cytoplasmic antibodies were also detected. The extractable nuclear antibody (ENA) panel showed positive results for anti-RNP (>644, normal range <20 CU), anti-SM (>694, normal range <20 CU), and SSA Ro60 (170, normal range <20 CU), as represented in Table 2. All myositis-specific antibodies and myositis-associated antibodies were negative, except for anti-Ku, as shown in Table 3. 

**Table 1:** Initial Laboratory Results Including Cardiac & Muscle Enzymes, Liver, and Renal Function Test

Test Category	Test	Patient Value	Reference Range	Units
Cardiac & Muscle Enzymes				
	Troponin T	673	< 15.0	ng/L
	Creatine Kinase (CK)	24,349	54 - 320	U/L
	Lactate Dehydrogenase (LDH)	1399	120 - 250	U/L
Liver Function Tests				
	Aspartate Aminotransferase (AST)	627	12 - 41	U/L
	Alanine Aminotransferase (ALT)	226	10 - 63	U/L
	Alkaline Phosphatase (ALP)	106	44 - 147	U/L
	Gamma-Glutamyl Transferase (GGT)	26	9 - 48	U/L
Renal Function Test				
	Creatinine	57	0.6 - 1.2	mg/dL

**Table 2:** Autoimmune Panel Showing Significant Autoimmune Markers ANA: Antinuclear Antibody dsDNA: Double-Stranded DNA RF: Rheumatoid Factor Sm: Smith Antigen RNP: Ribonucleoprotein SSA: Sjögren’s Syndrome Antigen A Ro52/TRIM21: Subtype of Ro antigen SSB: Sjögren’s Syndrome Antigen B

Test	Patient Value	Reference Range	Units
ANA	Positive	Negative	
ANA Pattern	Speckled		
ANA Titre	1:2560	< 1:80	Titre
Cytoplasmic Antibodies	Positive	Negative	
Anti-dsDNA	<10	< 30	IU/mL
RF	<10	< 14	IU/mL
Anti-Sm	>694	< 7.0	U/mL
Anti-Ro/SSA	170	< 20	U/mL
Anti-Ro52/TRIM21	<2	< 20	U/mL
Anti-La/SSB	<3	< 20	U/mL
Anti- RNP	>644	< 20	U/mL

**Table 3:** Myositis-Specific and Myositis-Associated Antibodies, and Scleroderma/Systemic Sclerosis Antibodies Jo-1: Histidyl tRNA Synthetase Mi-2: Nuclear Helicase Protein SRP: Signal Recognition Particle PL-7 and PL-12: Specific enzymes targeted in autoimmune myositis MDA5: Melanoma Differentiation-Associated Protein 5 TIF1-γ: Transcriptional Intermediary Factor 1-gamma NXP2: Nuclear Matrix Protein 2 Ku: DNA-Binding Protein involved in non-homologous end joining CN1A: Cytosolic 5'-Nucleotidase 1A HMG-CoA: 3-Hydroxy-3-Methylglutaryl-CoA Reductase Scl-70 (Topoisomerase I): Enzyme involved in DNA replication, targeted in scleroderma CENP A/B: Centromere Proteins A and B RP11: RNA Polymerase III Antibody Fibrillarin: Protein involved in ribosome synthesis NOR 90: Nucleolar Organizer Region 90 TH/TO: Small Nucleolar Ribonucleoproteins PM/Scl-100 and PM/Scl-75: Proteins targeted in overlap syndromes with polymyositis/scleroderma PDGFR: Platelet-Derived Growth Factor Receptor

Test Category	Test	Patient Value	Reference Result	Units
Myositis-Specific and Myositis-Associated Antibodies				
	Anti-Jo-1	Negative	Negative	
	Anti-Mi-2	Negative	Negative	
	Anti-SRP (Signal Recognition Particle)	Negative	Negative	
	Anti-PL-7	Negative	Negative	
	Anti-PL-12	Negative	Negative	
	Anti-MDA5	Negative	Negative	
	Anti-TIF1-γ	Negative	Negative	
	Anti-NXP2	Negative	Negative	
	Anti-Ku	Positive	Negative	
	Anti-CN1A Antibody	Negative	Negative	
	Anti-HMG-CoA Reductase Antibodies	<2	< 20	U/mL
Scleroderma/Systemic Sclerosis Antibodies				
	Anti-Scl-70 (Anti-Topoisomerase I)	Negative	Negative	
	Anti-CENP A	Negative	Negative	
	Anti-CENP B	Negative	Negative	
	Anti-RNA Polymerase III (RP11)	Negative	Negative	
	Anti-Fibrillarin	Negative	Negative	
	Anti-NOR 90	Negative	Negative	
	Anti-TH/TO	Negative	Negative	
	Anti-PM/Scl-100	Negative	Negative	
	Anti-PM/Scl-75	Negative	Negative	
	Anti-PDGFR	Negative	Negative	

**Figure 2:**
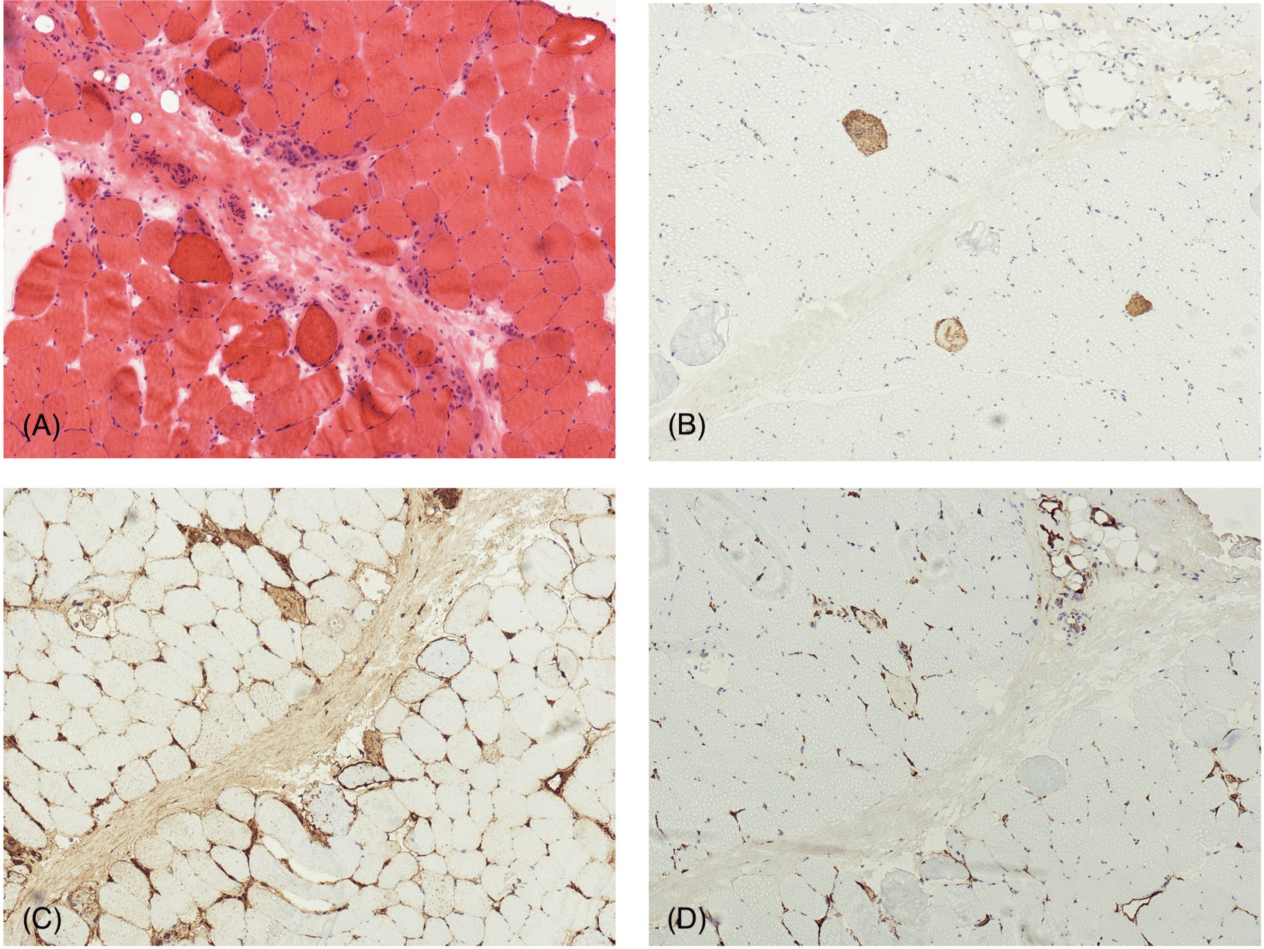
A) Perifascicular atrophy and regeneration of muscle fibers with expansion and fragmentation of perimysial connective tissue. B) Non-specific staining of necrotic fibers for the membrane attack complex. C) MHC1: Some sarcolemmal staining of perifascicular fibers for MHC1. D) No specific staining for MHC2. Diagnosis: Immune myopathy with perimysial pathology, suspicious for overlap myositis.

The diagnosis of overlap myositis was established based on these findings. Muscle biopsy findings were consistent with this diagnosis. The pathologist specifically commented on features consistent with scleromyositis, as demonstrated in Figure 2.

The patient underwent initial treatment with daily pulsed steroid therapy with methylprednisone at a dosage of 500 mg for five consecutive days. This was then followed by oral prednisone dosed at 1 mg/kg/day, followed by a prolonged taper. Intravenous immunoglobulin (IVIG) was administered at a dosage of 2 grams per kilogram over a period of two days every month. Rituximab was given intravenously at a dose of 1000 mg and then repeated after a two-week interval. After a period of two months, the patient experienced a 50% reduction in his CK levels. However, despite this improvement, the patient still required high doses of prednisone. Mycophenolate mofetil (MMF) was added to his regimen. This resulted in a notable reduction in CK and troponin levels, which returned to normal within a period of two months. We gradually reduced the prednisone dosage to a level below 15 mg daily. In the context of worsening neutropenia, we ultimately discontinued MMF. Following a 10-week period, the patient's muscle strength returned to normal. His chest discomfort and dyspnea resolved. Additionally, a repeated cardiac MRI showed no evidence of myocarditis seven months after the initial presentation.

## Discussion

In most cases, patients with anti-Ku antibodies are diagnosed with undifferentiated connective tissue disease (UCTD) or overlap syndromes, including polymyositis, SSc, and SLE. These patients often present with Raynaud's phenomenon, along with arthritis, myositis, and interstitial lung disease (ILD) [3,4]. Our case differs, though, in that the patient initially showed signs of symptomatic myocarditis and then developed proximal muscle weakness along with high-titer anti-RNP and anti-Ku antibodies. The incidence of myocarditis in patients with positive anti-Ku antibodies is not fully established. One meta-analysis found that 23% of people with anti-Ku antibodies had cardiac involvement, including myocarditis, all of whom were diagnosed with systemic sclerosis [5]. We could not identify evidence that anti-Ku antibodies directly increase the risk of myocarditis. Moreover, while ILD is commonly observed in patients with positive anti-Ku antibodies and autoimmune connective tissue disorders, especially in those with increased CK levels, the chest CT of our patient revealed no signs of ILD. Furthermore, he did not exhibit Raynaud's phenomenon, scleroderma skin signs, or sicca symptoms [6,7].

Anti-Ku antibody-associated myositis has been reported with variable patterns of muscle involvement. Some reported cases have described axial involvement, while in others, distal muscle involvement was more common [8,9]. In our case, only the proximal muscle groups were weak. The patient also had decreased reflexes, and his EMG showed both myotonic and myopathic discharges. These findings posed an initial diagnostic challenge, as without yet a muscle biopsy or autoantibody profile, his presentation may have been attributed to colchicine toxicity. Ultimately, the muscle biopsy did not show features consistent with colchicine-induced toxic vacuolar myopathy [10]. Instead, it demonstrated findings suggestive of overlap myositis. Histopathology revealed scattered, non-perifascicular necrotic fibers without significant lymphocytic infiltrates, pointing towards autoimmune necrotizing myositis (ANM). However, patients with anti-Ku antibodies, particularly those with overlap syndromes, may occasionally exhibit similar features. Another potential diagnosis is scleromyositis, a rising variant of myositis that presents with signs and symptoms of both SSc and myositis. Recent literature describes scleromyositis as more than an overlap syndrome, establishing it as a distinct clinical entity with unique clinical, serological, and histopathological features. However, no definitive diagnostic criteria currently exist for scleromyositis; diagnosis continues to depend on clinical observations and histological patterns validated by expert assessment [11].

A muscle biopsy from our patient showed perifascicular MHC I staining, a finding suggestive of scleromyositis rather than immune-mediated necrotizing myopathy (IMNM) [12,13]. Our patient's histopathology did not demonstrate capillary pathology characterized by prominent basement membrane reduplication, which is another distinguishing feature of scleromyositis. Although case series have reported scleromyositis without this feature, research on this aspect remains limited [14]. Ultimately, we determined the diagnosis to be MCTD with overlap myositis, given the patient's high anti-RNP antibody titers and clinical presentation with myositis and synovitis, but without key features of systemic sclerosis.

A study involving 34 patients with anti-Ku antibodies diagnosed with inflammatory myositis or overlap disease found that those without myositis or ILD generally required corticosteroid doses below 15 mg/d [15]. Another case report detailed a patient with polymyositis who tested positive for both anti-Ku and anti-Ro52 antibodies, had perimyocarditis, and experienced significant improvement with corticosteroids and MMF treatment [16]. Additionally, a case series involving three patients with limited scleroderma overlapping with myositis and myocarditis required a combination of multiple immunomodulatory therapies. Methotrexate (MTX) or MMF combined with IVIG were insufficient to control myocardial inflammation, necessitating the use of rituximab [17].

In our case, the patient required multiple lines of therapy, including pulsed IV steroids followed by high-dose oral steroids, monthly IVIG, rituximab, and MMF. The relationship between anti-Ku antibodies and myocarditis remains uncertain, particularly regarding any prognostic impact they may have. Rituximab proved effective in controlling our patient’s symptoms, consistent with its use in refractory myocarditis cases. It was well tolerated in our patient. 

## Conclusions

We are reporting a rare case of a young man diagnosed with MCTD and overlap myositis characterized by myocardial and proximal muscle involvement with high-titer anti-Ku and anti-RNP antibodies. Our patient required multiple lines of immunosuppressive therapy to achieve disease control. Further investigations are necessary to determine the prognosis and optimal treatment of patients with anti-Ku autoantibodies and myocarditis.
